# Supplementary material for: Tailored topotactic chemistry unlocks heterostructures of magnetic intercalation compounds
Source: Nat Commun. 2025 Jan 31;16:1208. doi: 10.1038/s41467-025-56467-9 (PMC11782516; doi:10.1038/s41467-025-56467-9)
Supplement: Supplementary file 1 — Supplementary Information [file 41467_2025_56467_MOESM1_ESM.pdf]

Supplementary Information for

Tailored topotactic chemistry unlocks heterostructures of magnetic intercalation compounds

Samra Husremović[1], Oscar Gonzalez[1], Berit H. Goodge[1,2], Lilia S. Xie[1], Zhizhi Kong[1], Wanlin Zhang[1], Sae Hee Ryu[3], Stephanie M. Ribet[4], Shannon S. Fender[1], Karen C. Bustillo[4], Chengyu Song[4], Jim Ciston[4], Takashi Taniguchi[5], Kenji Watanabe[6], Colin Ophus[7], Chris Jozwiak[3], Aaron Bostwick[3], Eli Rotenberg[3], and D. Kwabena Bediako[1,8,*]

[1]*Department of Chemistry, University of California, Berkeley, CA, 94720, USA*
[2]*Max-Planck-Institute for Chemical Physics of Solids, Nöthnitzer Str. 40, 01187, Dresden, Germany*
[3]*Advanced Light Source, Lawrence Berkeley National Laboratory, Berkeley, CA, 94720, United States*
[4]*National Center for Electron Microscopy, Molecular Foundry, Lawrence Berkeley National Laboratory, Berkeley, CA, USA*
[5]*Research Center for Functional Materials, National Institute for Materials Science, Tsukuba 305-0044, Japan*
[6]*International Center for Materials Nanoarchitectonics, National Institute for Materials Science, Tsukuba 305-0044, Japan*
[7]*Department of Materials Science and Engineering, Stanford University, Stanford, CA, 93405, USA*
[8]*Chemical Sciences Division, Lawrence Berkeley National Laboratory, Berkeley, CA 94720, USA*
[*]*Correspondence to: bediako@berkeley.edu*

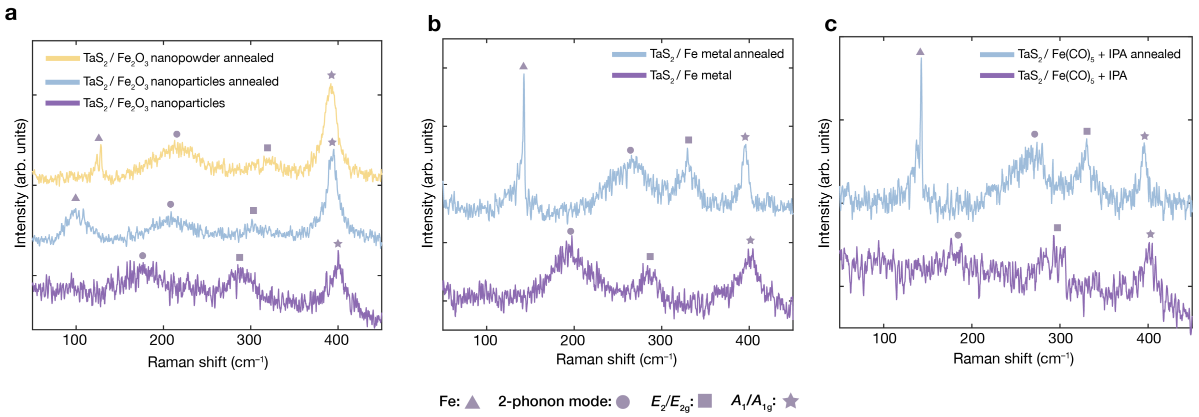

Supplementary Figure 1. Raman spectra of 2*H*–TaS_2 upon annealing in contact with different Fe precursors. (a–c) Change in the Raman spectra of 2*H*–TaS_2 upon annealing to 350 °C in contact with Fe_2O_3 nanoparticles/nanopowder **(a)**, evaporated Fe **(b)**, $Fe_xC_yO_z$ precursor deposited from $Fe(CO)_5$/isopropanol. Raman symmetry labels are added in (a–c) according to the legend outlined below the spectra.

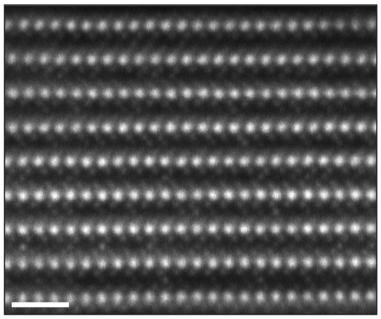

Supplementary Figure 2. Atomic-resolution imaging of sample S2. HAADF-STEM of sample S2, a $2H$–TaS_2 flake immersed in $Fe(CO)_5$/acetone for 24 hours at 48 °C and subsequently annealed to 200 °C for 4 hours. Scale bar: 1 nm.

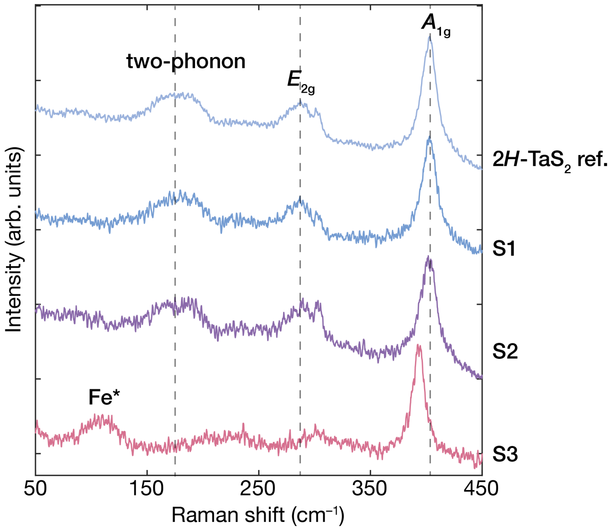

Supplementary Figure 3. Raman of samples S1–S3. Raman of samples S1–S3 and pristine, reference $2H$–TaS_2 flake that was not chemically treated. Samples S1–S3 were treated with $Fe(CO)_5$ in acetone and subsequently vacuum annealed at 100 °C, 200 °C and 350 °C, respectively. Thickness of S1–S3 is 12 nm, 7.8 nm, and 23 nm, respectively. S3 is the only sample whose Raman spectra exhibit Fe-related modes and peak shifts in comparison to the pristine sample.

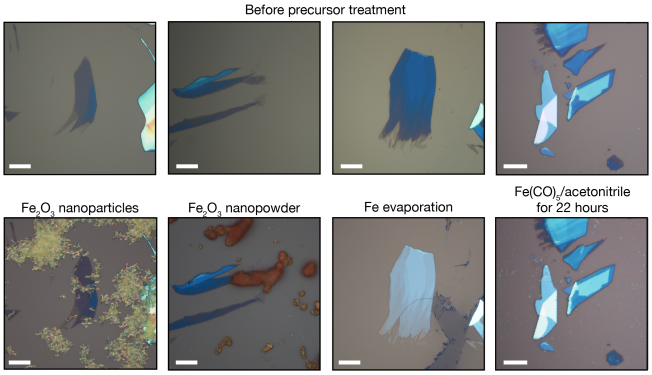

Supplementary Figure 4. Treatment of $2H$–TaS_2 flakes with different Fe precursors. Optical micrographs of $2H$–TaS_2 flakes before and after treatment with various Fe precursors. Scale bars: 10 μm.

Supplementary Note 1: Compositional and structural analysis of $Fe_xC_yO_z$ precursors

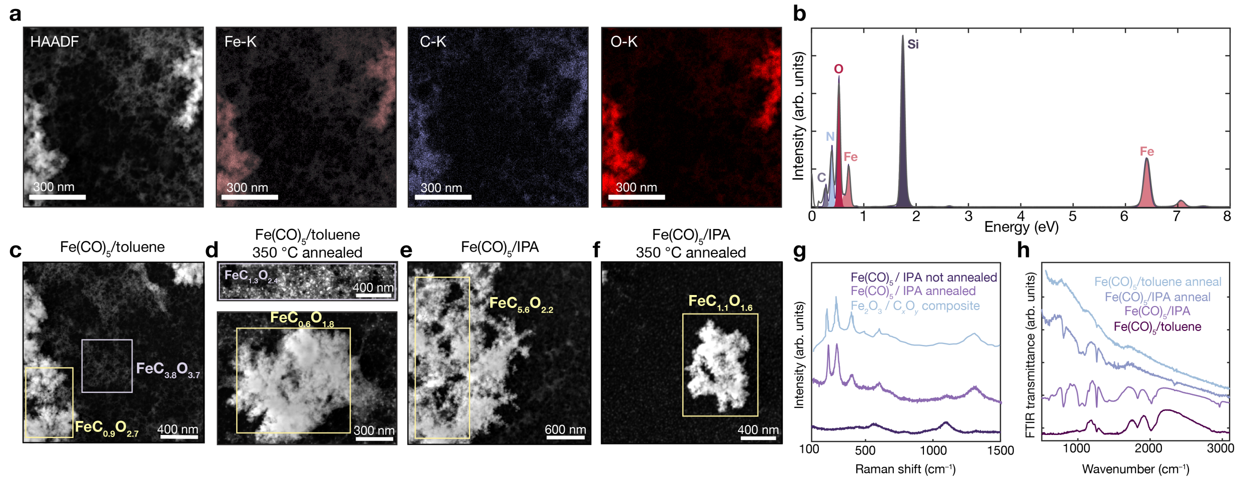

Supplementary Figure 5. Structural and compositional analysis of $Fe_xC_yO_z$ precursors. **(a)** Scanning transmission electron microscopy coupled with energy-dispersive X-ray spectroscopy (STEM-EDS) of $Fe_xC_yO_z$ made by drop casting a 370 mM solution of $Fe(CO)_5$/toluene onto a 30 nm silicon nitride TEM membrane. **(b)** Cumulative STEM-EDS spectrum of the sample from (a). **(c–f)** high-angle annular dark-field STEM (HAADF-STEM) images with marked composition of the indicated regions for $Fe_xC_yO_z$ prepared by drop-casting $Fe(CO)_5$/toluene **(c)**, drop-casting $Fe(CO)_5$/toluene followed by 350 °C annealing **(d)**, drop-casting $Fe(CO)_5$/isopropanol **(e)** and drop-casting $Fe(CO)_5$/isopropanol followed by annealing at 350 °C **(f)**. Samples were prepared on a 30 nm silicon nitride TEM membrane. **(g)** Raman spectra of as-prepared and annealed $Fe_xC_yO_z$ bulk powders alongside a reference spectrum of a composite comprising crystalline Fe_2O_3 and an amorphous carbon-oxygen polymer (C_xO_y) from reference [1]. **(h)** Fourier transform infrared (FTIR) spectra of $Fe_xC_yO_z$ bulk powders made with different solvents and annealing conditions.

Depositing a solution of $Fe(CO)_5$ onto solid substrates results in a composite $Fe_xC_yO_z$ material comprising particle and film components with varying Fe, C, and O ratios (Supplementary Figure 5a–f). Higher concentrations of $Fe(CO)_5$ tend to facilitate the formation of larger particle agglomerates. STEM-EDS analysis of the carbonyl-derived composites reveals Fe, C, and O are present in a molar ratio of approximately 1:3.5:3 and 1:1:2 before and after annealing, respectively (Supplementary Figure 5a–f), indicating a loss of C and O upon annealing. Additionally, we discover that the Raman spectra of annealed $Fe_xC_yO_z$ powders match those of crystalline Fe_2O_3 enclosed by an amorphous polymeric shell containing C and O (Supplementary Figure 5g). Evidence of a chemical change upon annealing is also evinced

by Fourier transform infrared (FTIR) spectroscopy of $Fe_xC_yO_z$ powders, with CO stretches around 2000 cm^{-1} suppressed post-annealing (Supplementary Figure 5h).

Supplementary Note 2: Oxidation state of Fe precursors

Fe oxidation states in various precursors were analyzed using energy-loss electron spectroscopy (EELS) and X-ray photoelectron spectroscopy (XPS), revealing distinct proportions of Fe^0, Fe^{2+}, and Fe^{3+}. Oxidized Fe species predominated across all samples, with only evaporated Fe films showing a minor proportion of Fe^0, amounting to less than 10 % of the total.

$Fe_xC_yO_z$ precursors contain a mixture of Fe^{3+} and Fe^{2+}, with the ratio influenced by the solvent used during film preparation. Solvents with higher oxygen and water content, such as acetone and isopropanol, produced $Fe_xC_yO_z$ with the highest proportion of Fe^{3+}. Specifically, we observed nearly 100 % Fe^{3+} using acetone (Figure 1e, Supplementary Figure 6) and 80 % Fe^{3+} using isopropanol (Supplementary Figure 7a,b). In contrast, films prepared in dry, degassed toluene contained about 60 % Fe^{3+} (Supplementary Figure 7c,d).

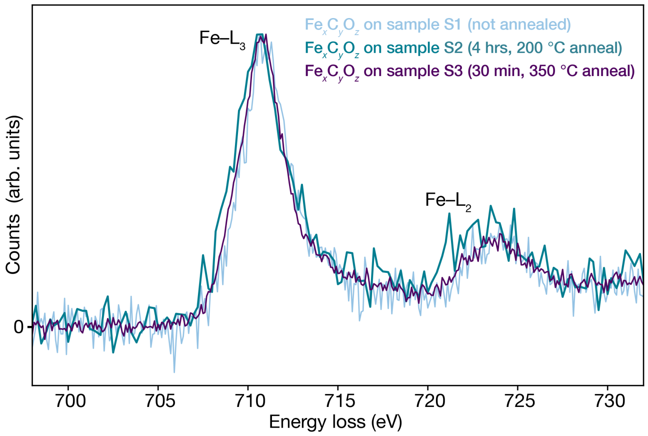

Supplementary Figure 6. EEL spectra of $Fe_xC_yO_z$ films formed on flakes S1–S3. Cumulative EEL spectra of the $Fe_xC_yO_z$ composite films that formed during the $Fe(CO)_5$/acetone treatment on top of S1–S3. Spectra are normalized by the L_3 peak maxima.

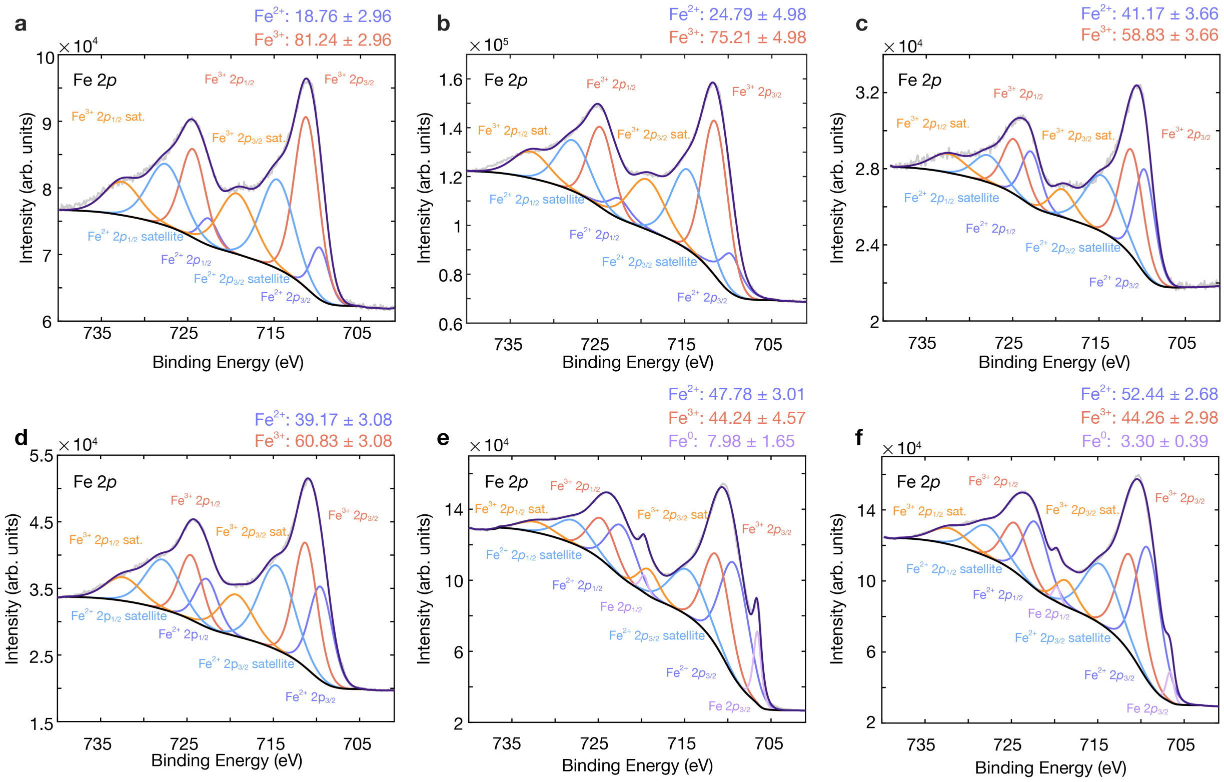

Supplementary Figure 7. X-ray photoelectron spectroscopy (XPS) of Fe precursors. (a–f) XPS spectra and corresponding curve fits are presented for $Fe_xC_yO_z$ prepared by drop-casting: 370 mM $Fe(CO)_5$/isopropanol **(a)**, 370 mM $Fe(CO)_5$/isopropanol with subsequent annealing at 350 °C **(b)**, 370 mM $Fe(CO)_5$/ toluene **(c)**, 370 mM $Fe(CO)_5$/toluene with subsequent annealing at 350 °C **(d)**, evaporated Fe **(e)**, and evaporated Fe with subsequent annealing at 350 °C **(f)**. $Fe_xC_yO_z$ precursors were prepared on Si substrates, whereas the evaporated Fe precursor was prepared on 90 nm SiO_2/Si. Ratios of measured Fe components are indicated above each spectrum.

Supplementary Note 3: Analysis of solid-state reactions

Solid state reaction between TaS_2 and Fe_2O_3 powders in a sealed ampoule (see Methods) forms products with three distinct crystallite colors/shapes as well as a yellow powder (Supplementary Figure 8a–d). Raman spectra of these products are consistent with the phonon modes of Fe_xTaS_2, Ta_2O_5, $1T$–TaS_2, and sulfur, respectively (Supplementary Figure 8e). The formation of products possessing compositions consistent with Ta_2O_5 and Fe_xTaS_2 from reaction of TaS_2 with Fe_2O_3 is validated by SEM-EDS analysis (Supplementary Figure 8f,g). Moreover, powder X-ray diffraction analysis (Supplementary Figure 9,10) supports a product mixture of Fe_xTaS_2, Ta_2O_5, $1T$–TaS_2, and $2H$–TaS_2 from these solid-state reactions.

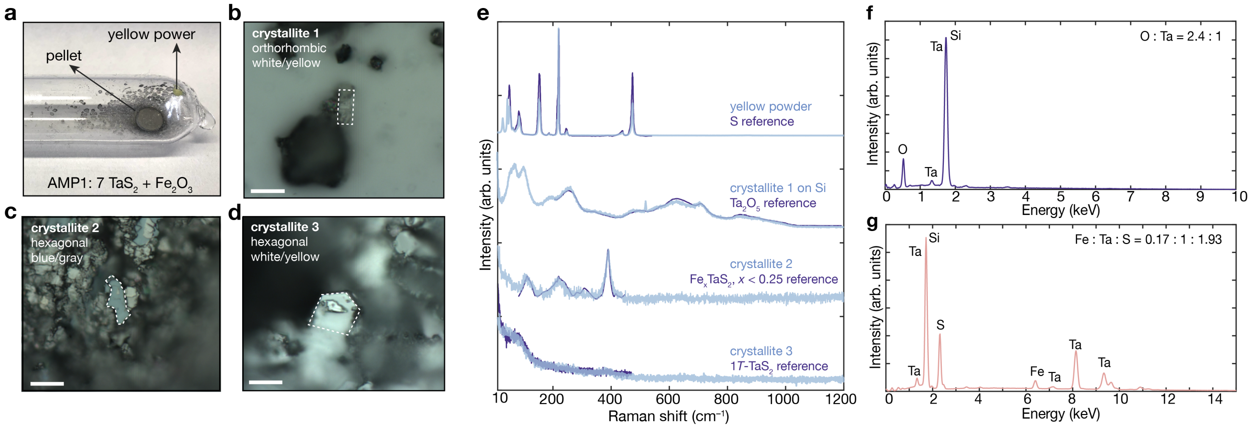

Supplementary Figure 8. Solid-state reactions of polycrystalline TaS_2 and Fe_2O_3. **(a)** Image of sealed glass ampoule enclosing a pellet made by mixing polycrystalline TaS_2 and Fe_2O_3 after a 7-day heating period at 900 °C. **(b–d)** Optical micrographs of three types of micron-sized crystallites obtained after crushing the pellet from (a). These crystallites, outlined in white dashed lines, display different optical contrasts and crystallographic symmetries. Scale bars (b–d): 10 μm. **(e)** Raman spectra of crystallites 1–3 from (b–d). Experimental data is overlaid with reference spectra for bulk $1T$–TaS_2 synthesized herein, and literature spectra for Fe_xTaS_2[2], Ta_2O_5[3] and S[4]. **(f–g)** Representative cumulative SEM-EDS spectra of crystallites whose Raman matches Ta_2O_5 **(f)** and Fe_xTaS_2 **(g)**. Crystallites were placed on a Si chip during the SEM-EDS acquisition.

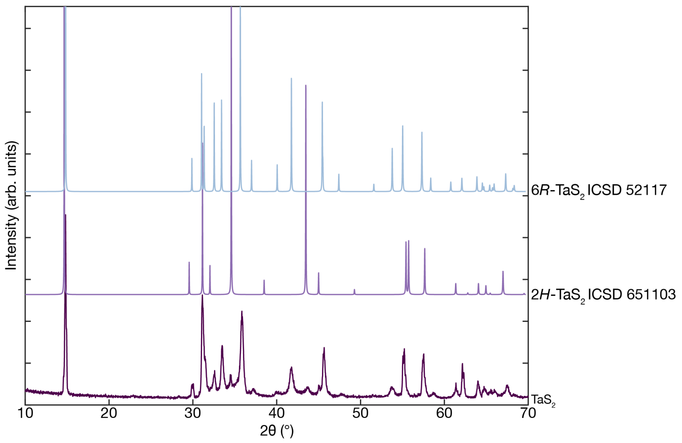

Supplementary Figure 9. Powder X-ray Diffraction (PXRD) pattern of polycrystalline TaS_2. Representative PXRD pattern of TaS_2 prepared by heating a stochiometric mixture of Ta (s) and S (s) to 900 °C for 7 days. Experimental data is plotted with literature spectra for $2H$–TaS_2[5] and $6R$–TaS_2[6]. The experimentally observed PXRD peaks present a combination of peaks from the plotted reference patterns.

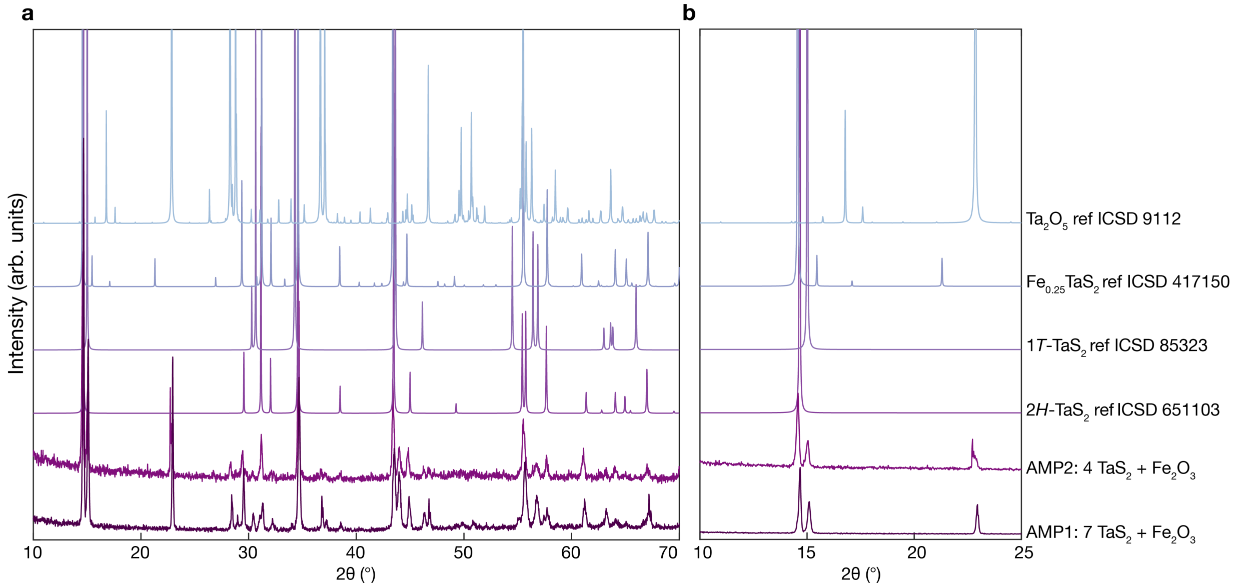

Supplementary Figure 10. **(a)** PXRD patterns after heating polycrystalline TaS_2 and Fe_2O_3 mixed in a 4:1 or 7:1 stoichiometric ratio for 7 days at 900 °C. Experimental data is plotted with literature patterns for $2H$–TaS_2[5], $1T$–TaS_2[7], $Fe_{0.25}TaS_2$[8] and Ta_2O_5[9]. The experimentally observed PXRD peaks present a combination of peaks from the plotted reference patterns. The presence of $1T$–TaS_2 (representing unreacted TaS_2) implies an incomplete solid-state reaction between TaS_2 and Fe_2O_3 following 7-day heating at 900 °C. **(b)** Diffraction patterns from (b) expanded in the range $2\theta = 10$ °– 25 °.

Supplementary Note 4: Solid-state reactions on the nanoscale

We examine the reactions of carbonyl-derived metal oxide films on nano-thick $2H$–TaS_2 flakes using high-resolution EELS (Supplementary Figure 11). These spectroscopic data show that $2H$–TaS_2 layers in immediate contact with carbonyl-derived Fe oxide films exhibit reduced crystallinity, which appears as blurring in the lattice images. This amorphization at the interface of $2H$–TaS_2 and $Fe_xC_yO_z$ is accompanied by a decreased S content and increased O content at the surface, but with crystalline Fe_xTaS_2 layers remaining underneath (Supplementary Figure 11).

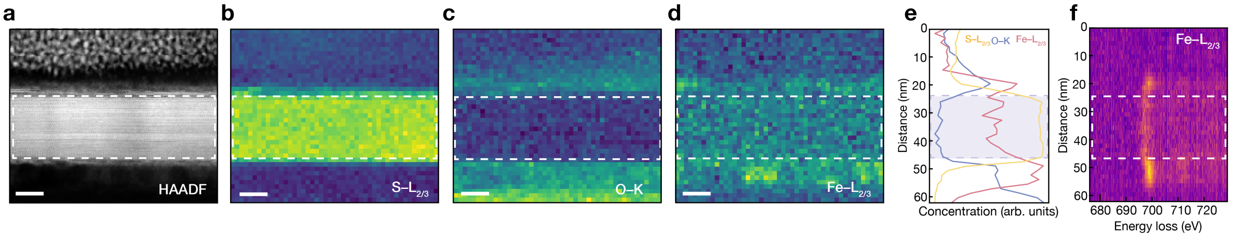

Supplementary Figure 11. Spatially resolved electron energy loss spectroscopy (EELS) of sample S3. Simultaneously recorded HAADF-STEM image of S3 **(a)** and S **(b)**, O **(c)** and Fe **(d)** elemental concentration maps. **(e)** Vertical line profile of the S, O, and Fe concentration. Scale bars (a–d): 10 μm. **(f)** Vertical profile of the Fe energy loss spectrum. The dashed white line in (a–d,f) and solid blue rectangle in (e) mark the crystalline flake region. Sample S3 was prepared by immersing $2H$–TaS_2 in $Fe(CO)_5$/acetone for 24 hours at 48 °C followed by annealing to 350 °C for 30 minutes. Delamination of the sample from the solid SiO_2/Si substrate coincided with the infiltration of $Fe_xC_yO_z$ beneath the sample; this was observed exclusively for samples prepared using $CaCl_2$-dried acetone.

Supplementary Note 5: Intercalation under hBN

We test the limits of intercalant diffusivity by partially encapsulating a flake of $2H$–TaS_2 with a thin crystal of hexagonal boron nitride, hBN, followed by treatment with the $Fe(CO)_5$ precursor and thermal annealing. STEM-EDS reveals that Fe is present both in $2H$–TaS_2 regions directly interfaced with the carbonyl-derived Fe oxide film and in the protected, hBN-covered sections (Supplementary Figure 12a). Furthermore, atomic-resolution HAADF-STEM imaging verifies that Fe intercalants occupy the pseudo-octahedral interstitial sites between $2H$–TaS_2 layers (Supplementary Figure 12b), while Raman spectroscopy evinces the ordered arrangement of the Fe intercalants (Supplementary Figure 12c).

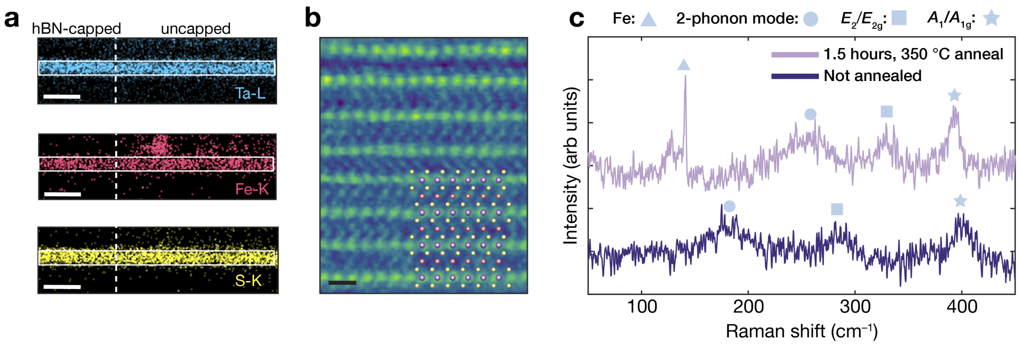

Supplementary Figure 12. Characterization of Fe intercalation in $2H$–TaS_2 partially encapsulated with hexagonal boron nitride (hBN). (a) STEM-EDS of a $2H$–TaS_2 flake that was partially encapsulated with hBN, treated with $Fe(CO)_5$/isopropanol, and annealed to 350 °C for 1.5 hours. The dashed white line separates the hBN-capped and uncapped regions, while the solid white line marks the flake border. Scale bars: 20 nm. **(b)** Atomic-resolution HAADF-STEM image of Fe_xTaS_2 formed in the hBN-encapsulated flake section of sample from (a). Crystal structure of $Fe_{1/3}TaS_2$ from ref [10] is overlaid with the experimental image. Scale bar: 5 Å. **(c)** Raman spectra of sample from (a) in the hBN-capped region before and after the vacuum annealing treatment. Raman symmetry labels are assigned according to the legend above the spectra.

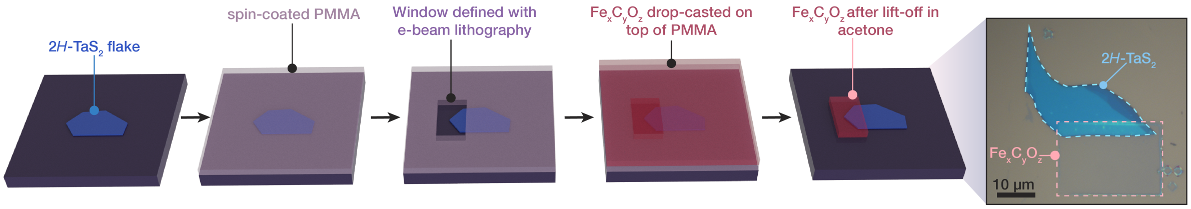

Supplementary Figure 13. Precursor patterning process. Schematic of the lithographic patterning process for $Fe_xC_yO_z$ with a representative optical micrograph of the final product.

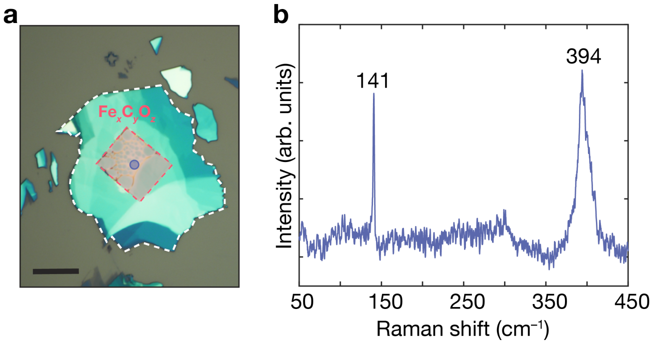

Supplementary Figure 14. Vertical diffusion of intercalants. (a) Optical micrograph of a $2H$–TaS_2 flake with a $Fe_xC_yO_z$ precursor patterned in the middle. The precursor was drop-casted from a 0.74 M solution of $Fe(CO)_5$ in isopropanol. The precursor is outlined in a red dashed line and false-colored red. The flake is outlined in a dashed white line. Scale bar: 10 μm. **(b)** Raman spectra of the sample from (a) in the region marked with a blue circle after annealing for 30 minutes at 250 °C. The Fe-related mode is evident at 141 cm^{-1}, and the A_{1g} mode is observed at 394 cm^{-1}; both spectral features are characteristic of intercalation.

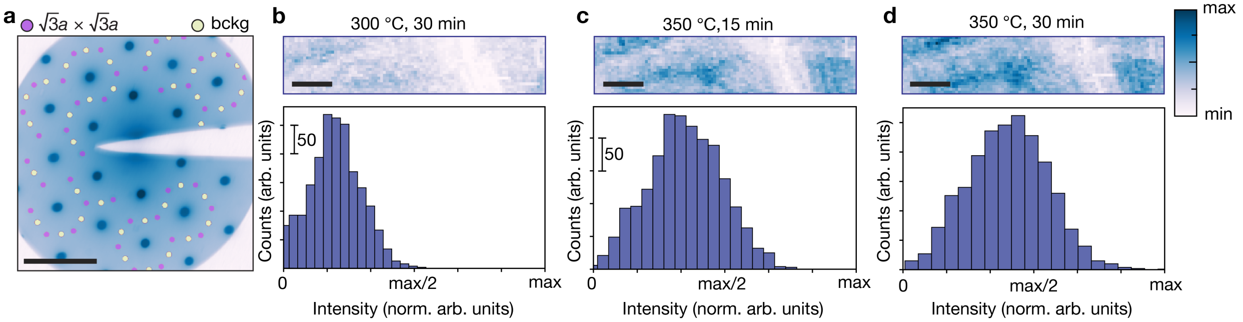

Supplementary Figure 15. Analysis of temperature-dependent four-dimensional scanning transmission electron microscopy (4D-STEM) data. (a) Mean diffraction pattern, displayed on a logarithmic scale, overlaid with virtual detectors (masks) for: $\sqrt{3}\times\sqrt{3}$ (purple) and background (yellow). Diffraction peaks for the 2×2 superlattice were not detected, informing placement of the background detector. Scale bar: 5 nm^{-1}. **(b–d)** Virtual dark-field images and their histograms obtained after in-situ annealing at 300 °C for 30 minutes **(b)**, 350 °C for 15 minutes **(c)**, and 350 °C for 30 minutes **(d)**. Data in (b–d) were normalized to the maximal value across the three datasets, and increasing intensities of superlattice peaks are correlated with increasing concentrations of ordered Fe intercalants. Scale bars (b–d): 200 nm.

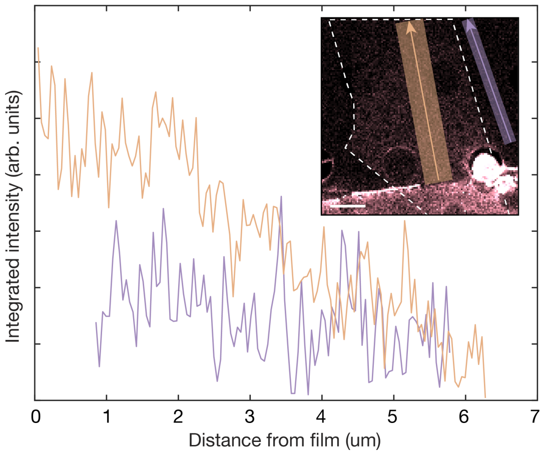

Supplementary Figure 16. Fe concentration profile for 2$\boldsymbol{H}$−TaS_2 annealed in contact with a patterned $Fe_xC_yO_z$ precursor. Averaged line profile of Fe extracted from the STEM-EDS map presented in Figure 2d. The dashed white line marks the perimeter of the flake. Line profiles in orange and violet were calculated for areas marked by their respective colors. Scale bar EDS map: 2 μm.

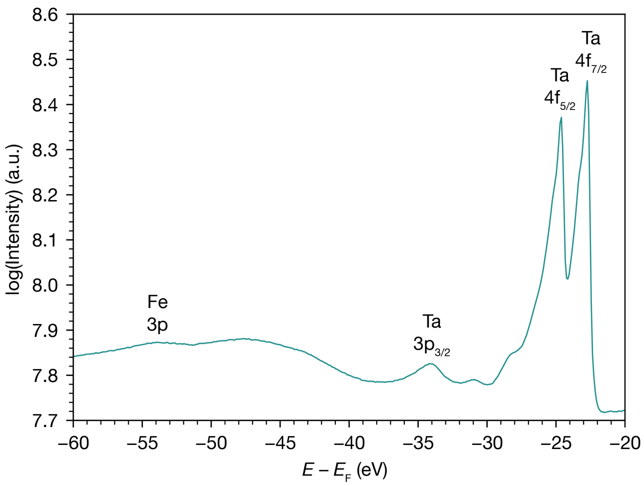

Supplementary Figure 17. Core-level spectra of the Fe_xTaS_2/hBN heterostructure. A representative core-level spectrum of the Fe_xTaS_2/hBN heterostructure measured with nano angle-resolved photoemission spectroscopy (nanoARPES). Data was obtained with $h\nu = 160$ eV at a 4 μm distance from the patterned $Fe_xC_yO_z$ precursor. Core level energies are referenced to the Fermi edge.

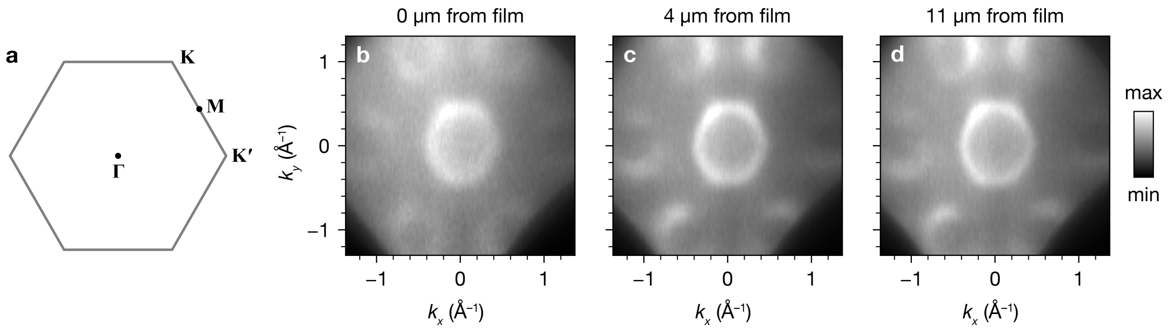

Supplementary Figure 18. Intensity distribution of Fermi surface cuts. (a) Schematic of the Brillouin zone for the primitive TaS_2 lattice. High-symmetry points are labeled. **(b–d)** Isoenergy cuts at $E = E_F$ measured for the Fe_xTaS_2/hBN heterostructure at 0 μm **(b)**, 4 μm **(c)** and 11 μm **(d)** away from the $Fe_xC_yO_z$ film. The intensity distribution changes from 6-fold symmetric in (b) to 3-fold symmetric in (c,d). Data is not normalized.

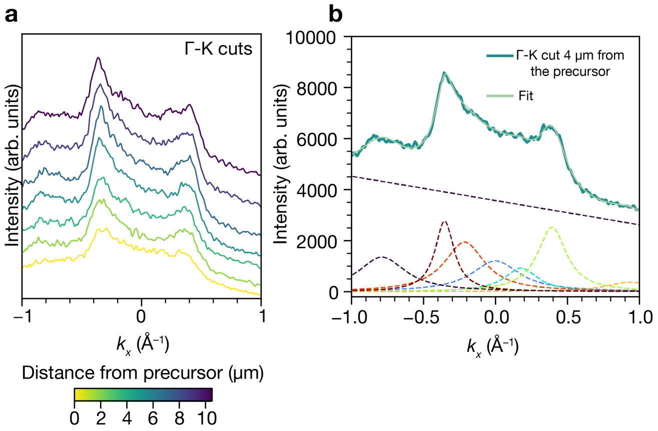

Supplementary Figure 19. Quantitative analysis of nano angle-resolved photoemission spectroscopic (nanoARPES) data. (a) Momentum distribution curves (MDCs) at the Fermi level (E_F) along Γ–K extracted for datasets obtained at different distances from the $Fe_xC_yO_z$ precursor. **(b)** Fitting a representative MCD curve to Lorentzians.

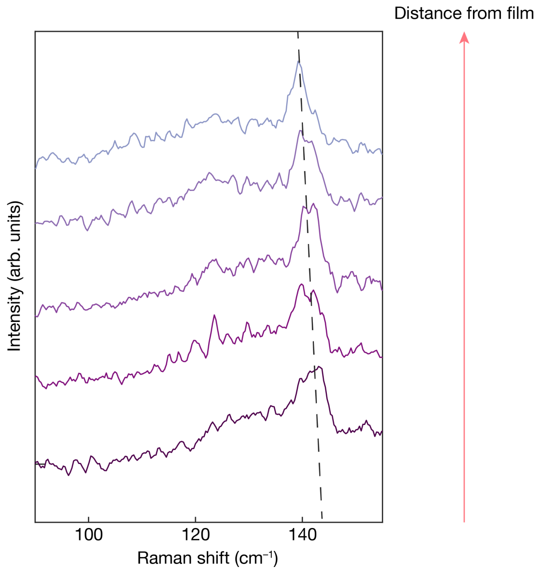

Supplementary Figure 20. Raman spectra of the sample measured with nanoARPES. Raman spectra of the nanoARPES sample measured at different distances from the $Fe_xC_yO_z$ precursor. Fe-related Raman modes of the nanoAREPS sample display red-shifting with increasing distance from the $Fe_xC_yO_z$ precursor, indicating a corresponding decrease in the Fe concentration.

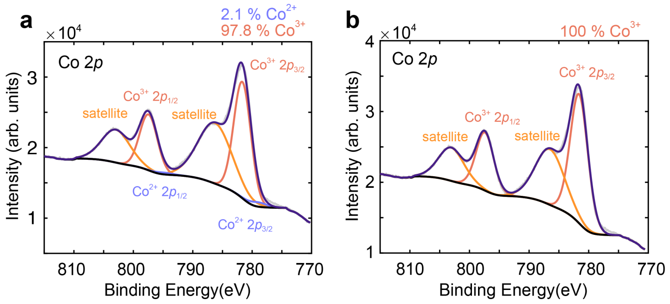

Supplementary Figure 21. XPS of the $Co_xC_yO_z$ precursor. **(a,b)** XPS spectra and corresponding curve fits are presented for $Co_xC_yO_z$ films prepared by drop-casting a solution of $Co_2(CO)_8$ in isopropanol **(a)** and toluene **(b)**.

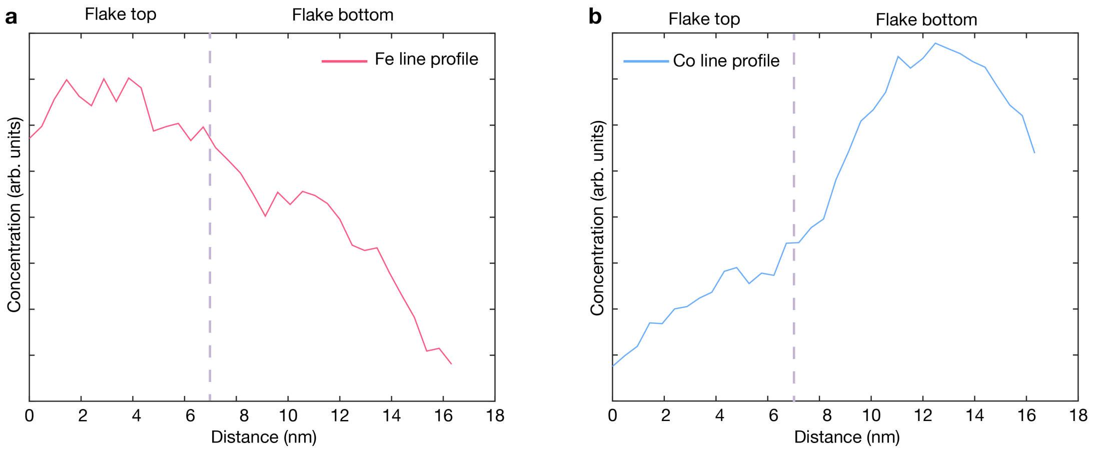

Supplementary Figure 22. STEM-EDS line profiles for the 2$\boldsymbol{H}$−TaS_2 heterostructure intercalated with Fe and Co. (a,b) Average line profiles of Fe **(a)** and Co **(b)** extracted from the STEM-EDS map presented in Figure 4h.

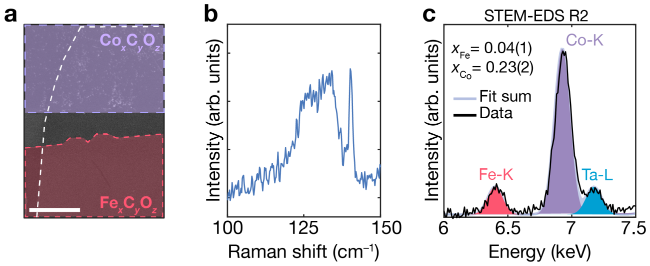

Supplementary Figure 23. Co-intercalation of Co and Fe in a singular 2*H*–TaS$_2$ flake. **(a)** Scanning electron microscopy (SEM) image of a 2H–TaS$_2$ flake (outlined with dashed white lines) with a Co$_x$C$_y$O$_z$ precursor patterned on the top (false-colored in violet) and a Fe$_x$C$_y$O$_z$ precursor patterned on the bottom (false-colored in red). Scale bar: 5 μm. **(b)** Low-frequency Raman spectrum of the flake region between the two metal precursors. **(c)** Cumulative STEM-EDS spectrum of the cross-sectioned flake region between the two metal precursors. Cross-sectioning was performed using a focused ion beam, and the STEM-EDS data was collected $\perp$ c axis of 2H–TaS$_2$.

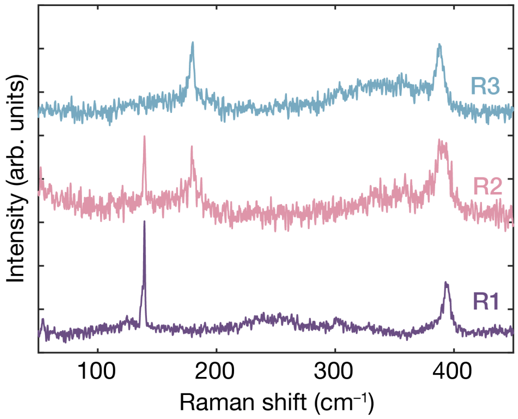

Supplementary Figure 24. Raman of device D1. Room-temperature Raman spectra of regions R1–R3 of device D1.

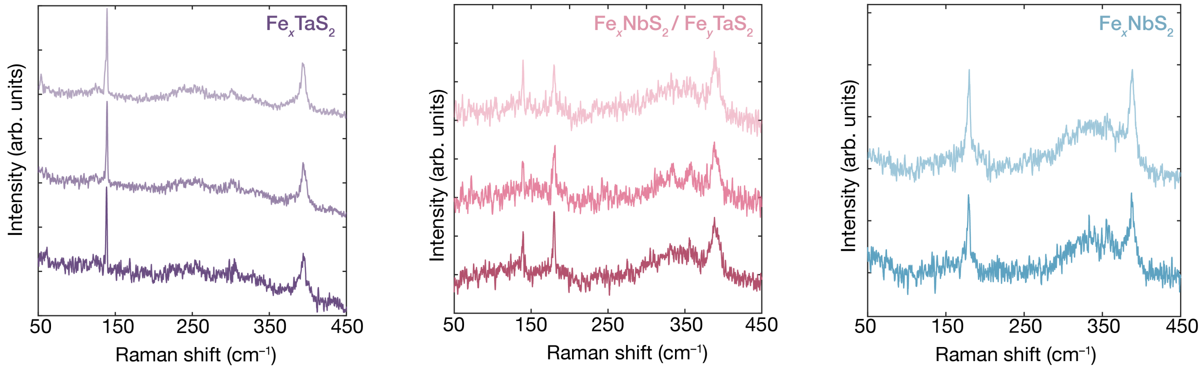

Supplementary Figure 25. Homogeneity of device D1. Raman spectra from different areas within regions R1–R3 of device D1.

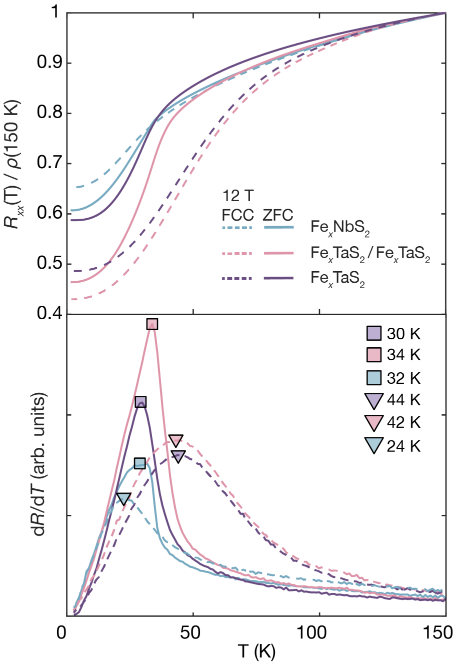

Supplementary Figure 26. Temperature-dependent resistance of device D1. Temperature-dependent resistance and its first derivative (dR/dT) for R1–R3 upon zero-field cooling (ZFC) and cooling in a 12 T magnetic field ($H \parallel c$). Maxima of dR/dT curves are labeled.

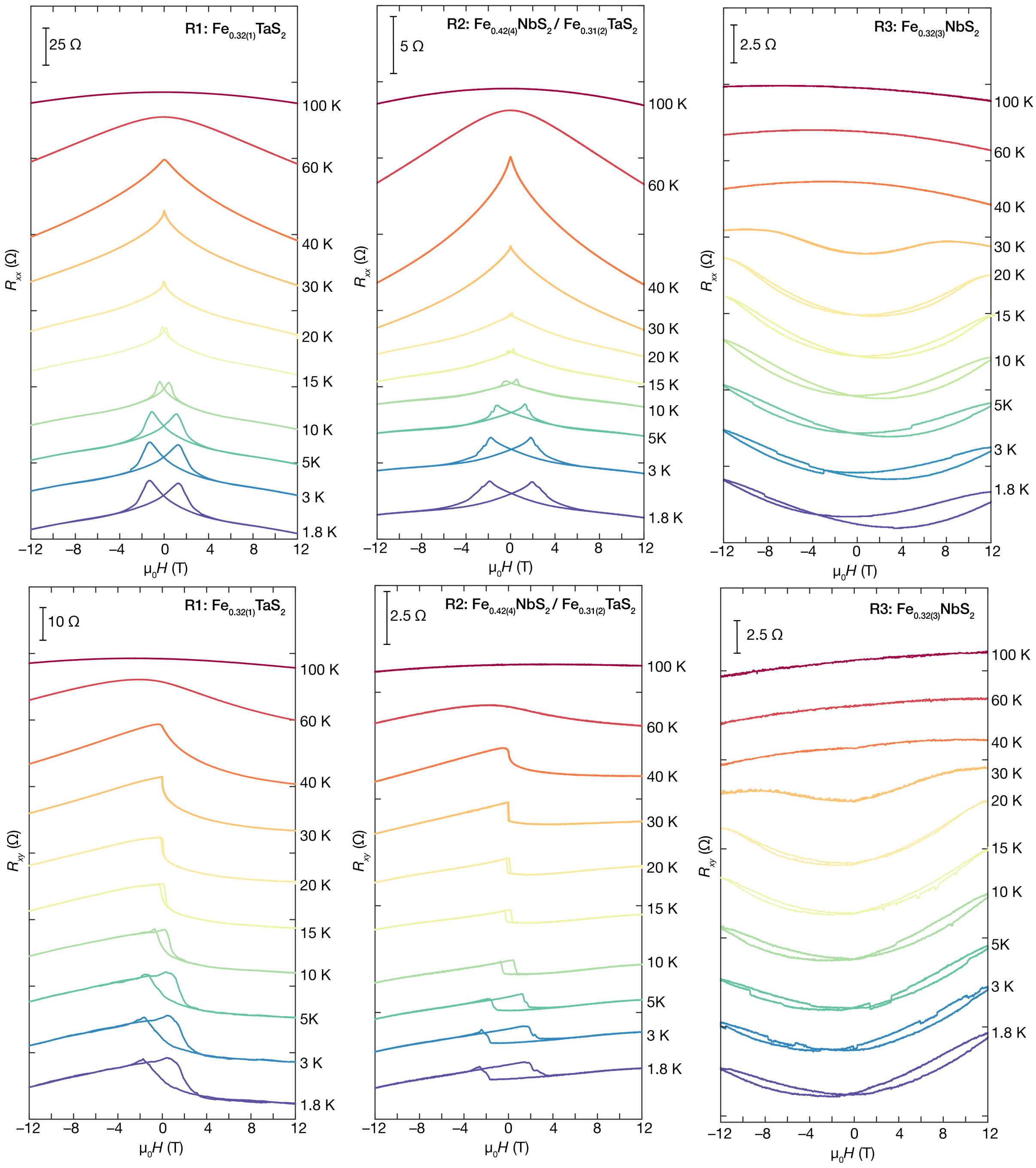

Supplementary Figure 27. Raw transport data for device D1. Raw data measured in regions R1–R3 of device D1. Data was obtained after the initial thermal and field cycling of the device.

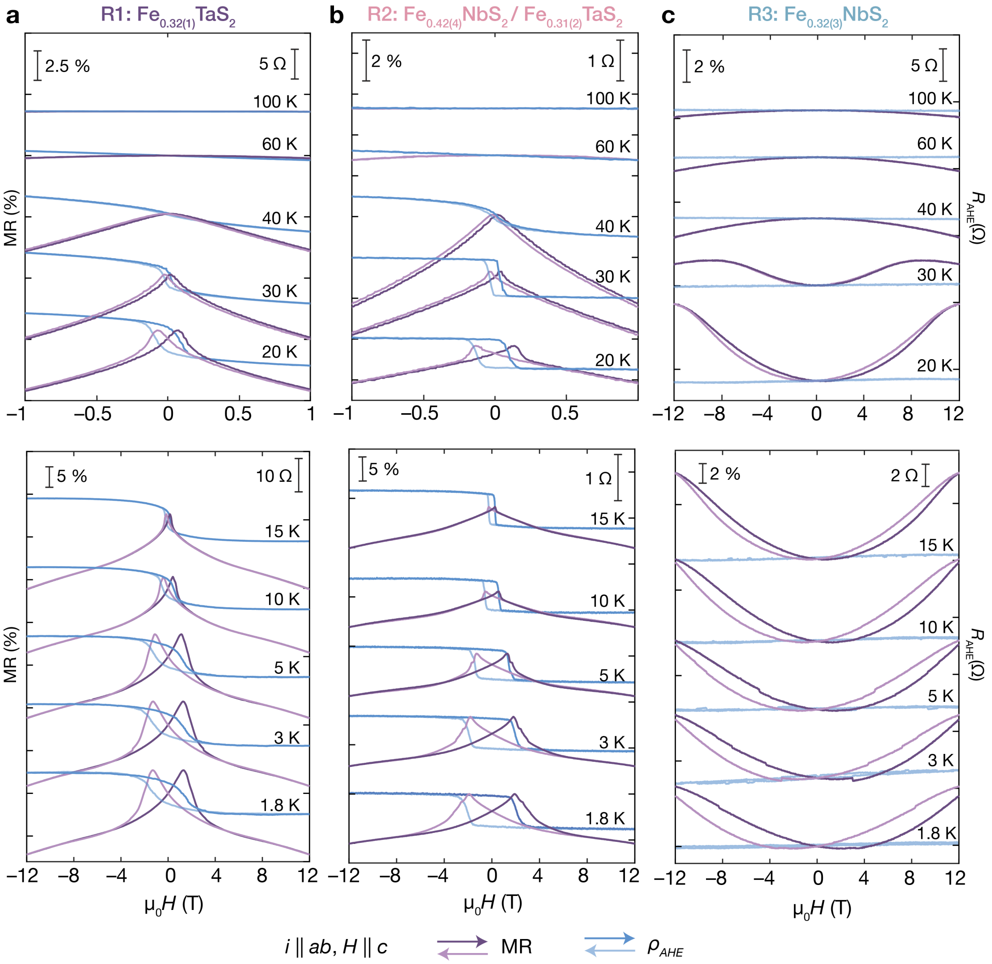

Supplementary Figure 28. Temperature-dependent magnetotransport of device D1. (a–c) Symmetrized temperature-dependent magnetoresistance (purple) and anomalous Hall resistance (blue) for R1 **(a)**, R2 **(b)** and R3 **(c)**. The stoichiometries of iron relative to the Ta/Nb stoichiometry were determined through STEM-EDS analysis of cross-sections prepared subsequent to magnetotransport experiments. Data was obtained after the initial thermal and field cycling of the device.

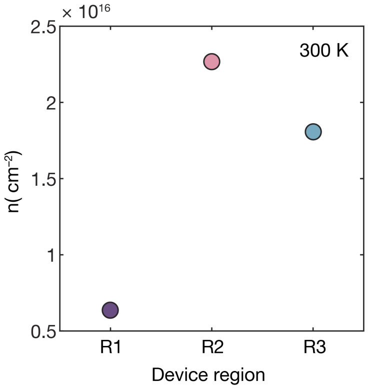

Supplementary Figure 29. Carrier density of device D1. Measured hole carrier density of device D1 in regions R1–R3 at 300 K.

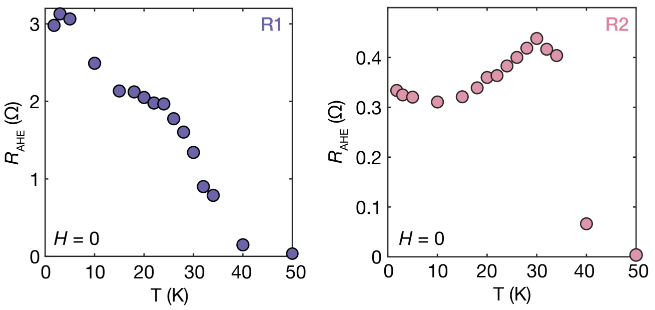

Supplementary Figure 30. Remnant anomalous Hall resistance for device D1. Temperature-dependence of the remnant anomalous Hall resistance R_{AHE} $(H = 0)$ for regions R1 and R2 of device D1.

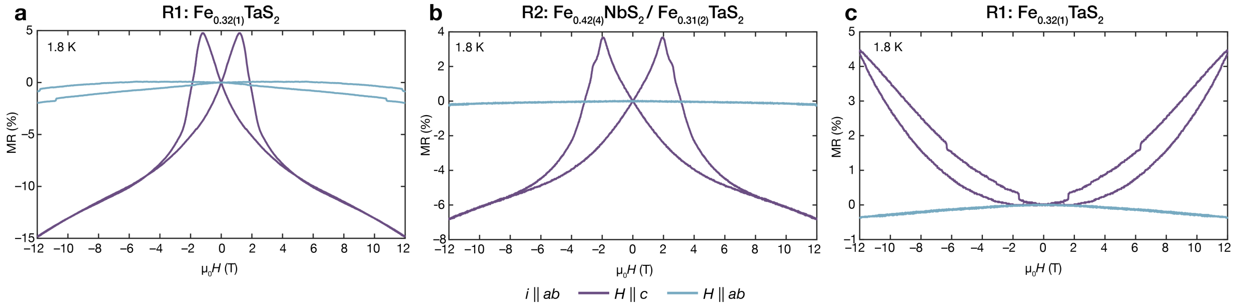

Supplementary Figure 31. Angle-dependent MR of device D1. (a–c) MR of device D1 with the external magnetic field aligned parallel and perpendicular to the *c*-axis for regions R1 **(a)**, R2 **(b), and R3 (c)**. During the measurements with $H \parallel ab$, the sample was rotated by approximately 4 degrees from the *ab*-axis, resulting in a non-zero field contribution in the $H \parallel c$ direction. The observed hysteresis in (a) with $H \parallel ab$ can be attributed to this misalignment.

Supplementary Note 6: Magnetic relaxation measured for device D1

To investigate magnetic relaxation in regions R1–R3 of device D1, we examine the changes in longitudinal resistance (R_{xx}) immediately after applying a magnetic field of 12 T (Supplementary Figure 32a). For R1 and R2, R_{xx} remains stable following magnetization (Supplementary Figure 32a). In contrast, R_{xx} of R3 evinces both gradual changes and abrupt jumps at 1.8 K (Supplementary Figure 32a,b), reflecting slow and rapid magnetic relaxation, respectively. Slow magnetic relaxation is typically linked to glassy magnetic behavior[11,12], while stochastic resistance jumps suggest the motion of pinned domain walls (DWs) driven by the applied field[13,14]. Stochastic resistance jumps are also prominent in isothermal field sweeps for R3 below 10 K (Supplementary Figures 33, 34). At higher temperatures, signatures of magnetic relaxation are not evident for R3 (Supplementary Figure 32b), consistent with the expected fast relaxation times and diminishing domain pinning at elevated temperatures.

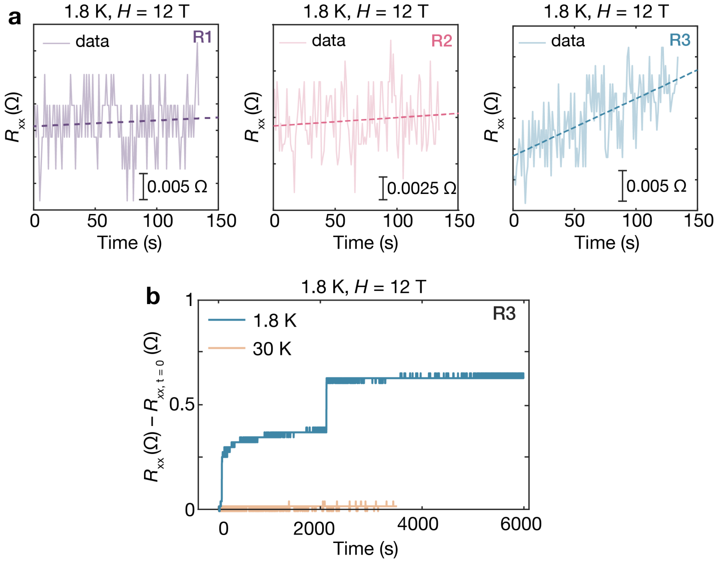

Supplementary Figure 32. Magnetic relaxation behavior of device D1. (a) Time-dependent longitudinal resistance (R_{xx}) of device regions R1–R3 at 12 T, immediately following a forward magnetic sweep from 0 T to 12 T at 1.8 K. Dashed lines in (a) serve as visual guides for the data trend. **(b)** Time-dependent change in the longitudinal resistance from the value recorded immediately after a forward magnetic sweep from −12 T to 12 T at 1.8 K and 30 K. For (a) and (b), $H \parallel c$ and $i \parallel ab$.

Examining magnetic relaxation is essential for understanding the R_{xx} hysteresis shape observed in isothermal field sweeps for R3. Specifically, this relaxation may cause the resistance states at 12 T to differ between forward and reverse sweeps, as shown in measurements 1, 5, and 6 in the Supplementary Figure 33a. In these cases, the sample was held at 12 T for over 30 minutes before the reverse sweep from 12 T to −12 T (data before the sweep was not recorded) and only 60 seconds after the forward sweep (−12 T to 12 T). This difference in hold times, and consequently in magnetic relaxation, leads to the variation in resistance

states at 12 T for different sweep directions. In contrast, for measurements 2–4, the sample was held at 12 T for 60 seconds before the reverse sweep and after the forward sweep, resulting in nearly identical resistance at 12 T for both directions (Supplementary Figure 33a). It is important to note that if resistance states differ at 12 T between sweeps, the hysteresis appears open (discontinuous) after the symmetrization procedure (Supplementary Figure 33b), which mathematically equates the sweep directions. Moreover, varying relaxation times at 12 T between sweeps cause vertical offsets, altering the crossing point between sweeps and leading to asymmetric hystereses (Supplementary Figure 33a). Such asymmetry is not seen in the raw data for R1 and R2, which do not exhibit magnetic relaxation (Supplementary Figure 35).

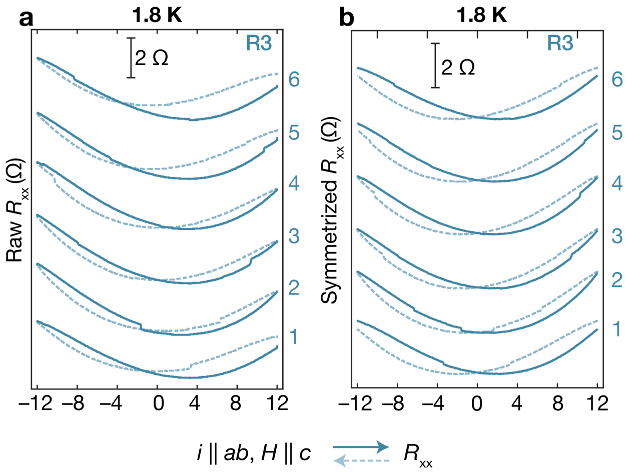

Supplementary Figure 33. Repeated measurements of field-dependent longitudinal resistance for region R3 of device D1 at 1.8 K. (a,b) Field-dependent raw **(a)** and symmetrized **(b)** longitudinal resistance (R_{xx}) of recorded for region R3 of device D1 at 1.8 K. Data from six measurements, labeled 1–6, is shown. Each measurement was obtained after ZFC protocol and started with a reverse sweep (12 T to −12 T) and ended in a forward sweep (−12 T to 12 T).

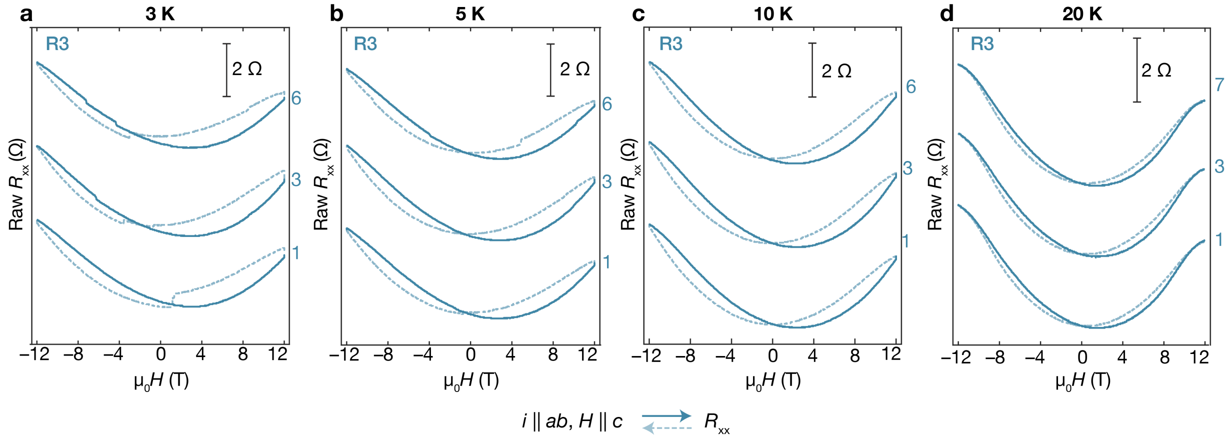

Supplementary Figure 34. Repeated measurements of field-dependent longitudinal resistance for region R3 of device D1 above 1.8 K. (a–d) Raw longitudinal resistance (R_{xx}) as a function of magnetic field for region R3 at 3 K **(a)**, 5 K **(b)**, 10 K **(c)**, and 20 K **(d)**. Each dataset, labeled 1, 3, and 6, shows measurements starting with a reverse field sweep (12 T to −12 T) and ending with a forward sweep (−12 T to 12 T). The sample was held at 12 T for 5 minutes before the reverse sweep and for 60 seconds after the forward sweep.

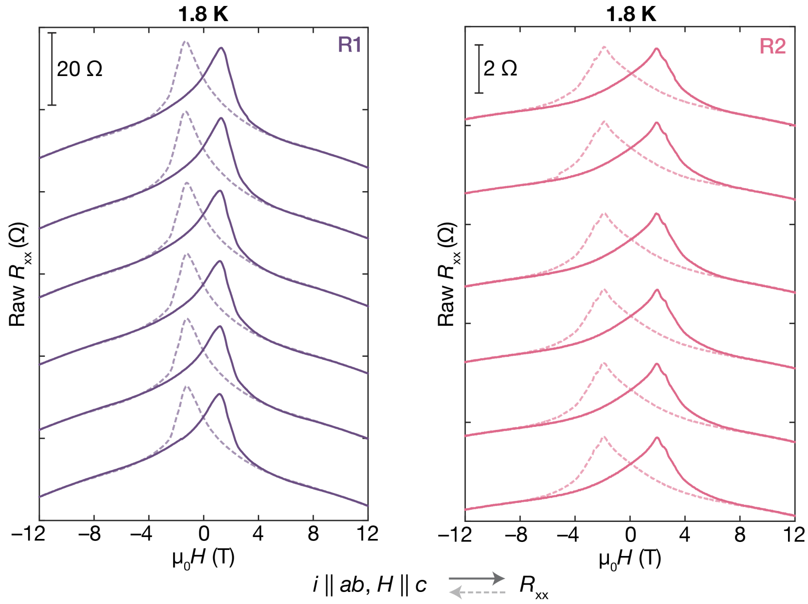

Supplementary Figure 35. Repeated measurements of field-dependent longitudinal resistance for regions R1 and R2 of device D1 at 1.8 K. (a,b) Raw field-dependent longitudinal resistance (R_{xx}) recorded for regions R1 **(a)** and R2 **(b)** 1.8 K. Data from six measurements is shown. Each measurement started with a reverse sweep (12 T to −12 T) and ended in a forward sweep (−12 T to 12 T).

Supplementary Note 7: Potential field-driven metamagnetic transitions

Field-driven metamagnetic transitions from an antiferromagnetic to a metastable ferromagnetic state have been reported in bulk Fe_xTiS_2, materials closely related to those used in device D1. These transitions can be detected by comparing initial magnetization curves after zero-field cooling with subsequent magnetic field sweeps. Large, irreversible changes in magnetoresistance indicate the presence of metamagnetic transitions[15–18]. In regions R1 and R2, the resistance of the initialization curves falls within the range recorded during subsequent field sweeps, suggesting no metamagnetic changes (Supplementary Figure 36) . However, in region R3, the resistance of the initialization curve lies outside this range Supplementary Figure 36), indicating possible metamagnetic and irreversible changes when a field is applied. The absence of anomalous Hall effect (Supplementary Figure 28c), suggests that the system does not transition to an ensemble ferromagnetic state after field sweeps. This hints that the field might be stabilizing a minority magnetic phase, such as a glassy uncompensated phase.

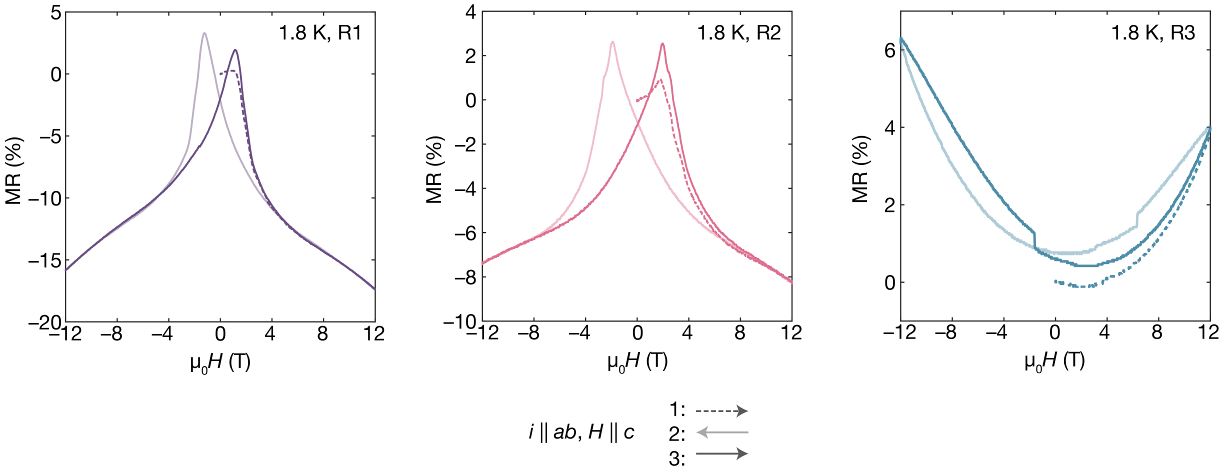

Supplementary Figure 36. Magnetoresistance including initialization curves. (a–c) Magnetoresistance data recorded for regions R1 **(a)**, R2 **(b)**, and R3 **(c)** of device D1 at 1.8 K. The initial magnetization was obtained after zero-field cooling (ZFC). Each measurement began with a forward initialization sweep (0 to 12 T), followed by a reverse sweep (12 T to −12 T), and concluded with a forward sweep (−12 T to 12 T). Data was not symmetrized and magnetoresistance was defined as: $MR(\%) = [(R_{xx}(H) - R_{xx,init}(H = 0)/R_{xx,init}(H = 0)] \times 100\%$, where $R_{xx,init}(H = 0)$ is the resistance of the initial magnetization curve at zero external magnetic field.

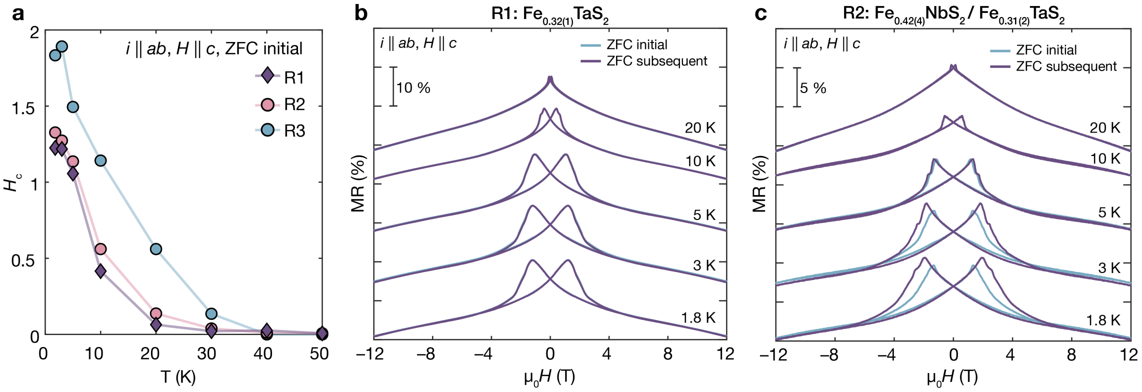

Supplementary Figure 37. Change in MR of device D1 after the initial thermal and field cycling. **(a)** Coercive fields (H_c) measured for regions R1–R3 of device D1 after the first cooling of the device. Data was obtained upon warming. **(b,c)** Temperature-dependent MR for R1 **(b)** and R2 **(c)** measured after the first zero-field cooling (ZFC) and subsequent ZFC protocols. Temperature-dependent H_c was unchanged for R1, while H_c for R2 increased following the first thermal and field cycling. This suggests a magnetostrictive process occurring in the R2 heterostructure region.

Supplementary References

1. Ryu, Y. J., Kim, M. & Yoo, C.-S. Phase diagram and transformations of iron pentacarbonyl to nm layered hematite and carbon-oxygen polymer under pressure. *Scientific reports* **5,** 15139 (2015).
2. Husremović, S. et al. Hard ferromagnetism down to the thinnest limit of iron-intercalated tantalum disulfide. *Journal of the American Chemical Society* **144,** 12167–12176 (2022).
3. Manukumar, K., Viswanatha, R. & Nagaraju, G. Ionic liquid–assisted hydrothermal synthesis of Ta_2O_5 nanoparticles for lithium-ion battery applications. *Ionics* **26,** 1197–1202 (2020).
4. Valerjevich, K. E., Alexeevich, S. V. & Viktorovich, E. A. Nanostructure of matrices for sulfur constructional composites: methodology, methods and research tools.
5. Johnston, D. & Keelan, B. Superconductivity and magnetism of $M_x(H_2O)_yTaS_2$ layered cointercalation compounds. *Solid State Communications* **52,** 631–634 (1984).
6. Jellinek, F. The system tantalum-sulfur. *Journal of the Less Common Metals* **4,** 9–15 (1962).
7. Spijkerman, A., de Boer, J. L., Meetsma, A., Wiegers, G. A. & van Smaalen, S. X-ray crystal-structure refinement of the nearly commensurate phase of $1T$–TaS_2 in (3+2)-dimensional superspace. *Physical review B* **56,** 13757 (1997).
8. Morosan, E. et al. Sharp switching of the magnetization in $Fe_{1/4}TaS_2$. *Physical Review B* **75,** 104401 (2007).
9. Stephenson, N. & Roth, R. Structural systematics in the binary system Ta_2O_5–WO_3. V. The structure of the low-temperature form of tantalum oxide L-Ta_2O_5. *Acta Crystallographica Section B: Structural Crystallography and Crystal Chemistry* **27,** 1037–1044 (1971).
10. Wijngaard, J., Hass, C. & Devillers, M. Optical and magneto-optical properties of $Fe_{0.28}TaS_2$. *Journal of Physics: Condensed Matter* **3,** 6913 (1991).
11. Kong, Z. et al. Near room-temperature intrinsic exchange bias in an Fe intercalated $ZrSe_2$ spin glass. *Journal of the American Chemical Society* **145,** 20041–20052 (2023).

12. Maniv, E. et al. Exchange bias due to coupling between coexisting antiferromagnetic and spin-glass orders. *Nature Physics* **17,** 525–530 (2021).

13. Muñoz, M. & Prieto, J. L. Suppression of the intrinsic stochastic pinning of domain walls in magnetic nanostripes. *Nature communications* **2,** 562 (2011).

14. Sugimoto, S. et al. Electrical nucleation, displacement, and detection of antiferromagnetic domain walls in the chiral antiferromagnet Mn3Sn. *Communications Physics* **3,** 111 (2020).

15. Baranov, N. et al. Magnetic order, field-induced phase transitions and magnetoresistance in the intercalated compound $Fe_{0.5}TiS_2$. *Journal of Physics: Condensed Matter* **25,** 066004 (2013).

16. Baranov, N. V. et al. Magnetic phase transitions, metastable states, and magnetic hysteresis in the antiferromagnetic compounds $Fe_{0.5}TiS_{2-y}Se_y$. *Phys. Rev. B* **100,** 024430 (2019).

17. Selezneva, N. V., Baranov, N. V., Sherokalova, E. M., Volegov, A. S. & Sherstobitov, A. A. Multiple magnetic states and irreversibilities in the Fe_xTiS_2 system. *Phys. Rev. B* **104,** 064411 (2021).

18. Selezneva, N. V., Sherokalova, E. M., Podlesnyak, A., Frontzek, M. & Baranov, N. V. Relationship between magnetoresistance behavior and magnetic states in intercalated compounds Fe_xTiS_2. *Phys. Rev. Mater.* **7,** 014401 (2023).
